# Single-nucleotide polymorphism-based genetic diversity analysis of the Kilakarsal and Vembur sheep breeds

**DOI:** 10.14202/vetworld.2017.549-555

**Published:** 2017-05-26

**Authors:** Rathinasamy Selvam, Nagarajan Murali, A. Kannan Thiruvenkadan, Ramesh Saravanakumar, Gurusamy Ponnudurai, Thilak Pon Jawahar

**Affiliations:** Department of Animal Genetics and Breeding, Veterinary College and Research Institute, Tirunelveli - 627 358, Tamil Nadu, India

**Keywords:** allele discrimination module, competitive allele-specific polymerase chain reaction, Kilakarsal, single-nucleotide polymorphism, Toll-like receptor genes, Vembur

## Abstract

**Aim:**

The present study was thus undertaken to analyze the genetic diversity of Kilakarsal and Vembur sheep breeds using single-nucleotide polymorphism (SNP) markers within Toll-like receptor (TLR) 3, 5, 6, 9, and 10 genes.

**Materials and Methods:**

Competitive allele-specific polymerase chain reaction (PCR)-based end-point genotyping was performed using real-time PCR to type the SNPs. Allele discrimination module implemented in real-time PCR was utilized to call the genotypes based on fluorescence intensity recorded for each of the two alleles. Basic diversity indices, namely, gene frequencies, observed heterozygosity, expected heterozygosity, and inbreeding coefficient (F_IS_), and testing for Hardy–Weinberg equilibrium (HWE) were estimated using package for elementary analysis of SNP data software program.

**Results:**

Of the 25 SNPs, 22 were found to be polymorphic, whereas two SNPs, namely, TLR3_1081_AC and TLR9_2036_CT, were monomorphic in both Kilakarsal and Vembur sheep populations. The SNP TLR10_1180_AG was monomorphic in Kilakarsal but polymorphic in Vembur sheep. The observed heterozygosities were estimated as 0.289 and 0.309 in Kilakarsal and Vembur sheep, respectively, whereas the expected heterozygosity values were 0.305 and 0.309 in the two breeds, respectively. The overall mean F_IS_ was 0.107 ranging from −0.005 to 0.241 in Kilakarsal sheep and −0.047 ranging from −0.005 to 0.255 in Vembur sheep. In Kilakarsal sheep, the test for HWE revealed TLR9_1308_GC SNP locus with significant deviation (p<0.05) due to heterozygosity deficit. In Vembur sheep, TLR10_82_CT and TLR10_292_CG loci showed significant deviation (p<0.05) due to heterozygosity excess. Other SNP loci did not deviate from HWE (p>0.05) revealing that the population was in HWE proportions.

**Conclusions:**

The SNP markers within five TLR genes (TLR3, TLR5, TLR6, TLR9, and TLR10) utilized for genotyping in this study were highly polymorphic in Kilakarsal and Vembur breeds of sheep. This study on the genetic diversity analysis of the Kilakarsal and Vembur sheep breeds revealed considerable genetic variation within the breeds and it can be utilized to improve desirable traits.

## Introduction

In India, sheep breeds are well-known for ability to withstand harsh climatic conditions, disease resistance, and lack of fodder. There has been a rapid decline in population and uniqueness of most of the indigenous sheep populations through breed substitution, indiscriminate crossbreeding, and the absence of conservation programs. At present, to meet the consumer demand and global climate change and emerging diseases, there is an urgent need to maintain the biodiversity and conservation of valuable native germplasm. Several studies have been carried out to characterize the sheep breeds based on morphometric characters, production, and reproduction performances [1]. Breed characterization requires basic knowledge of genetic variations that can be effectively measured within and between populations. Previous studies focused on genetic diversity and population structures were based on morphological markers, chromosomal karyotyping, and biochemical markers. Recently, due to the remarkable progress in the field of molecular biology, new class of markers called DNA molecular markers had been discovered, for example, restriction fragment length polymorphism, random-amplified polymorphic DNA, amplified fragment length polymorphism, single-strand conformation polymorphism, mitochondrial DNA, and microsatellite markers [2].

However, at present, single-nucleotide polymorphism (SNP) is becoming the standard marker for diversity analysis and genome-wide studies. The usefulness of SNPs in analyses of population diversity and structure has been demonstrated in several studies [3]. SNP is a single base change in a DNA sequence. Advantages of the SNP markers such as located in coding area of DNA, stably inherited than other DNA markers, and more suited as long-term selection make SNP markers as a powerful new tool for genetic selection. SNPs are more suitable than microsatellites for high-throughput genetic analysis. Due to their extensive distribution and abundant variations, SNPs play an important role in livestock population structure, genetic variation, origin, and evolution research [4].

There are many SNP techniques that can be used depending on the purpose of the research considering throughput, data turnaround time, ease of use, performance, flexibility, requirements, and cost. For a small number of SNPs, a uniplex assay like KBioscience competitive allele-specific polymerase chain reaction (PCR) genotyping system (KASP) is used. The KASP method is more cost-effective than multiplex methods. There is also a much shorter turnaround time to receive the results with the KASP method than other multiplex methods. In addition, there is a lower genotyping error rate of 0.7-1.6%. The KASP method is more flexible than other methods in that it can be used when there are many SNPs in a few samples or when there are few SNPs in many samples [5].

The present study was thus undertaken to analyze the genetic diversity of Kilakarsal and Vembur sheep breeds using SNP markers within Toll-like receptor (TLR) 3, 5, 6, 9, and 10 genes through competitive allele-specific PCR. Many studies have reported that genetic variation in the TLR genes modifies cellular immune response and alters susceptibility to disease. Recent studies have indicated that there were plenty of polymorphisms in the TLR genes in humans and livestock [6]. This study will be of immense use in identifying genetic structure of these breeds and for planning the organized breeding program for their genetic improvement, formulating effective conservation strategies for genetic diversity within breeds, and sustainable utilization.

## Materials and Methods

### Ethical approval

This research was conducted in sheep breeds with natural infection only. Hence the ethical approval is not required.

### Selection of animals

**Figure-1:**
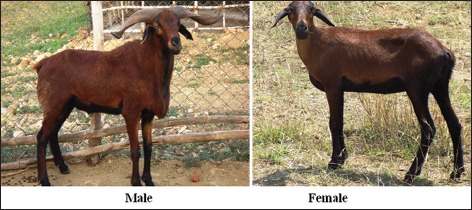
Kilakarsal sheep

**Figure-2:**
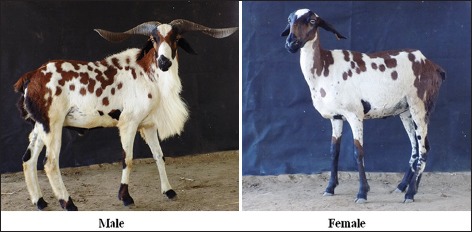
Vembur sheep

This study was carried out in Kilakarsal sheep (Figure-1) maintained at Instructional Livestock Farm Complex, Veterinary College and Research Institute, Tirunelveli, Tamil Nadu, India, and District Livestock Farm, Abishekappatti, Tirunelveli, Tamil Nadu, India, and Vembur sheep (Figure-2) maintained at Instructional Livestock Farm Complex, Veterinary College and Research Institute, Tirunelveli, and Government Sheep Farm, Sattur, Virudhunagar, Tamil Nadu, India. A total of 100 sheep, 50 each of Kilakarsal and Vembur sheep breeds belongs to the both sexes, were selected randomly.

### DNA isolation and purification

The volume of 5 ml of blood was collected routinely from all the animals from external jugular vein using vacutainer. All the chemicals and reagents used in this experiment were of molecular biology grade and obtained from Hi-Media and Sigma-Aldrich. DNA was isolated from blood samples using a modified high salt method [7]. The purity and concentration of DNA samples were checked by 1% agarose gel electrophoresis Nanodrop (Thermo Scientific).

### Competitive allele-specific PCR-based end point genotyping

Competitive allele-specific PCR-based end-point genotyping was performed using real-time PCR to type the SNPs. Allele discrimination module implemented in real-time PCR was utilized to call the genotypes based on fluorescence intensity recorded for each of the two alleles. The genotyping system KASP is a homogeneous, fluorescent, end-point genotyping technology. KASP genotyping assays are based on competitive allele-specific PCR and enable bi-allelic scoring of SNPs and insertions and deletions at a specific locus. Details of SNP name of the gene, chromosome location, alleles at each locus, genic region, synonyms/non-D synonyms, and amino acid change are presented in Table-1.

**Table-1:** Detail of different TLR gene SNP loci examined in this study.

SNP	Gene name	Chromosome	Strand	Alleles	Genic region	Synonyms/non-synonyms	AA change
TLR3_1081_AC	TLR3	26	+	A/C	CDS	Non-synonyms	A-Ile; C-Leu
TLR3_265_CT	TLR3	26	+	C/T	CDS	Non-synonyms	C-Arg; T-Trp
TLR3_340_CT	TLR3	26	+	C/T	CDS	Non-synonyms	C-Arg; T-Cys
TLR3_370_AG	TLR3	26	+	A/G	CDS	Non-synonyms	A-Asn; G-Asp
TLR3_631_AG	TLR3	26	+	A/G	CDS	Non-synonyms	A-Arg; G-Gly
TLR5_1354_AG	TLR5	12	-	A/G	CDS	Non-synonyms	A-Lys; G-Glu
TLR5_1578_CT	TLR5	12	-	C/T	CDS	Synonyms	CT-Asp
TLR5_2037_CT	TLR5	12	-	C/T	CDS	Synonyms	CT-Tyr
TLR5_276_CT	TLR5	12	-	C/T	CDS	Synonyms	CT-Ser
TLR5_786_CT	TLR5	12	-	C/T	CDS	Synonyms	CT-Ser
TLR6_1301_AG	TLR6	6	-	A/G	CDS	Non-synonyms	A-Met; G-Val
TLR6_229_GT	TLR6	6	-	G/T	CDS	Non-synonyms	G-Met; T-Ile
TLR6_49_CT	TLR6	6	-	C/T	CDS	Synonyms	CT-Phe
TLR6_589_AG	TLR6	6	-	A/G	CDS	Synonyms	AG-Thr
TLR6_814_AC	TLR6	6	-	A/C	CDS	Non-synonyms	A-Glu; C-Asp
TLR9_1308_GC	TLR9	19	+	G/C	CDS	Non-synonyms	G-Gly; C-Arg
TLR9_1769_CT	TLR9	19	+	C/T	CDS	Synonyms	CT-Val
TLR9_2036_CT	TLR9	19	+	C/T	CDS	Synonyms	CT-Cys
TLR9_2099_CT	TLR9	19	+	C/T	CDS	Synonyms	CT-Ser
TLR9_2504_CT	TLR9	19	+	C/T	CDS	Synonyms	CT-Asn
TLR10_1180_AG	TLR10	6	-	A/G	CDS	Non-synonyms	A-Ile; G-Val
TLR10_292_CG	TLR10	6	-	C/G	CDS	Non-synonyms	C-Leu; G-Val
TLR10_595_AG	TLR10	6	-	A/G	CDS	Non-synonyms	A-Ile; G-Val
TLR10_771_CT	TLR10	6	-	C/T	CDS	Synonyms	CT-Leu
TLR10_82_CT	TLR10	6	-	C/T	CDS	Synonyms	CT-Leu

TLR=Toll-like receptor, SNP=Single-nucleotide polymorphism

### Equipment used

The quantitative real-time PCR machine (ILLUMINA, USA, Eco™ Real-Time PCR System, Catalog # EC-900-1001) was utilized for KASP genotyping assay.

### Statistical analysis

Basic diversity indices, namely, gene frequencies, observed heterozygosity, expected heterozygosity, and inbreeding coefficient (F_IS_), and testing for Hardy–Weinberg equilibrium (HWE) were estimated using package for elementary analysis of SNP data software program [8].

## Results

### The gene frequency

Allele frequency of SNP loci at different TLR genes in Kilakarsal and Vembur sheep breeds of Tamil Nadu has been tabulated (Table-2). Of the 25 SNPs, 22 were found to be polymorphic, whereas two SNPs, namely, TLR3_1081_AC and TLR9_2036_CT, were monomorphic in both Kilakarsal and Vembur sheep populations. The SNP TLR10_1180_AG was monomorphic in Kilakarsal but polymorphic in Vembur sheep. In the polymorphic SNP loci, the allele frequency ranged from 0.08 to 0.92 in Kilakarsal sheep and 0.03 to 0.97 in Vembur sheep and the allele frequency ranged from 0.07 to 0.93 in overall.

**Table-2:** Allele frequency at different TLR gene SNP loci in Kilakarsal and Vembur sheep.

SNP_ID	Kilakarsal		Vembur		Overall	
		
	Allele 1	Allele 2	Allele 1	Allele 2	Allele 1	Allele 2
TLR3_265_CT	0.47	0.53	0.37	0.63	0.42	0.58
TLR3_340_CT	0.76	0.24	0.80	0.20	0.78	0.22
TLR3_370_AG	0.24	0.76	0.20	0.80	0.22	0.78
TLR3_631_AG	0.24	0.76	0.12	0.88	0.18	0.82
TLR3_1081_AC	0.00	1.00	0.00	1.00	0.00	1.00
TLR5_1354_AG	0.28	0.72	0.31	0.69	0.30	0.71
TLR5_1578_CT	0.45	0.55	0.51	0.49	0.48	0.52
TLR5_2037_CT	0.72	0.28	0.70	0.30	0.71	0.29
TLR5_276_CT	0.91	0.09	0.97	0.03	0.94	0.06
TLR5_786_CT	0.28	0.72	0.32	0.68	0.30	0.70
TLR6_1301_AG	0.19	0.81	0.30	0.70	0.25	0.76
TLR6_229_GT	0.90	0.10	0.95	0.05	0.93	0.07
TLR6_49_CT	0.10	0.90	0.05	0.95	0.07	0.93
TLR6_589_AG	0.90	0.10	0.95	0.05	0.93	0.07
TLR6_814_AC	0.91	0.09	0.95	0.05	0.93	0.07
TLR9_1308_GC	0.02	0.98	0.67	0.33	0.35	0.66
TLR9_1769_CT	0.99	0.01	0.86	0.14	0.93	0.07
TLR9_2036_CT	0.00	1.00	0.00	1.00	0.00	1.00
TLR9_2099_CT	0.66	0.34	0.71	0.29	0.69	0.32
TLR9_2504_CT	0.36	0.64	0.34	0.66	0.35	0.65
TLR10_1180_AG	0.00	1.00	0.01	0.99	0.01	1.00
TLR10_292_CG	0.38	0.62	0.44	0.56	0.41	0.59
TLR10_595_AG	0.08	0.92	0.04	0.96	0.06	0.94
TLR10_771_CT	0.92	0.08	0.96	0.04	0.94	0.06
TLR10_82_CT	0.38	0.62	0.44	0.56	0.41	0.59

TLR=Toll-like receptor, SNP=Single-nucleotide polymorphism

### Heterozygosity

The observed and expected heterozygosity at different TLR gene SNP loci in Kilakarsal and Vembur sheep are furnished in Table-3. The observed heterozygosities were estimated as 0.289 and 0.309 in Kilakarsal and Vembur sheep, respectively, whereas the expected heterozygosity values were 0.305 and 0.309 in the two breeds, respectively. The estimated mean values were found to be more than 0.50 in TLR3_265_CT and TLR5_1578_CT in Kilakarsal sheep and TLR3_265_CT, TLR5_1578_CT, and TLR9_1308_GC in Vembur sheep, indicating high levels of within-population diversity. However, these diversity indices were estimated to be low in other SNP loci studied in the Kilakarsal and Vembur sheep.

**Table-3:** Observed heterozygosity, expected heterozygosity, and inbreeding coefficient at different TLR gene SNP loci in Kilakarsal and Vembur sheep.

SNP_ID	Kilakarsal			Vembur		
	
	Ho	He	F_IS_	Ho	He	F_IS_
TLR3_265_CT	0.500	0.503	0.001	0.500	0.471	−0.068
TLR3_340_CT	0.360	0.368	0.018	0.240	0.323	0.255
TLR3_370_AG	0.360	0.368	0.018	0.240	0.323	0.255
TLR3_631_AG	0.360	0.368	0.018	0.240	0.213	−0.131
TLR5_1354_AG	0.400	0.407	0.013	0.460	0.432	−0.070
TLR5_1578_CT	0.500	0.500	−0.005	0.500	0.505	0.005
TLR5_2037_CT	0.400	0.407	0.013	0.440	0.424	−0.043
TLR5_276_CT	0.140	0.165	0.150	0.060	0.059	−0.026
TLR5_786_CT	0.400	0.407	0.013	0.480	0.440	−0.098
TLR6_1301_AG	0.300	0.311	0.030	0.440	0.424	−0.043
TLR6_229_GT	0.160	0.182	0.116	0.100	0.096	−0.048
TLR6_49_CT	0.160	0.182	0.116	0.100	0.096	−0.048
TLR6_589_AG	0.160	0.182	0.116	0.100	0.096	−0.048
TLR6_814_AC	0.140	0.165	0.150	0.100	0.096	−0.048
TLR9_1308_GC	0.000	0.040	1.000	0.500	0.447	−0.126
TLR9_1769_CT	0.020	0.020	−0.005	0.200	0.243	0.174
TLR9_2099_CT	0.400	0.453	0.114	0.380	0.416	0.082
TLR9_2504_CT	0.440	0.465	0.050	0.480	0.453	−0.065
TLR10_1180_AG	0.000	0.000	nd	0.020	0.020	−0.005
TLR10_292_CG	0.360	0.476	0.241	0.720	0.498	−0.457
TLR10_595_AG	0.120	0.149	0.190	0.080	0.078	−0.037
TLR10_771_CT	0.120	0.149	0.190	0.080	0.078	−0.037
TLR10_82_CT	0.360	0.476	0.241	0.720	0.498	−0.457
Overall	0.289	0.305	0.107	0.309	0.309	−0.047

Ho=Observed heterozygosity, He=Expected heterozygosity, F_IS_=Inbreeding coefficient, nd=Not deducted, TLR=Toll-like receptor, SNP=Single-nucleotide polymorphism

### Inbreeding coefficient (F_IS_)

The F_IS_ value at each locus notably varied in different loci (Table-3). The overall mean F_IS_ was 0.107, and it varied from −0.005 to 0.241 in Kilakarsal sheep breed and −0.047 (−0.005-0.255) in Vembur sheep. The size and sign of F_IS_ reflect the deviation from HWE of the genotypes such that when F_IS_ is zero the locus is in HWE, and when F_IS_ is positive, there is a deficiency in heterozygotes. Negative F_IS_ values indicate that the level of heterozygosity is higher than its expectation from HWE [9]. The average F_IS_ in each sheep population of small size was very low. Studies have found that small populations exhibit high inbreeding. Some F_IS_ values were high, for example, in Kilakarsal SNP locus, TLR10_292_CG and TLR10_82_CT showed a value of 0.241, and in Vembur SNP locus, the TLR3_340_CT and TLR3_370_AG showed a value of 0.255. This reflects a high frequency of a particular allele among homozygotes.

The estimated F_IS_ values across 20 SNP loci were found to be positive in Kilakarsal sheep, except for TLR5_1578_CT and TLR9_1769_CT, whereas, in Vembur sheep population, the estimated F_IS_ values were found to be positive only in TLR3_340_CT, TLR3_370_AG, TLR5_1578_CT, TLR9_1769_CT, and TLR9_2099_CT loci. Positive values for F_IS_ indicate decrease of heterozygous in the population and negative F_IS_ values reveal an increase in heterozygosity.

### Testing for HWE

The numbers of SNP loci deviating from HWE at different TLR gene SNP loci in Kilakarsal and Vembur sheep are presented in Table-4. The heterozygosity deficit ranged from 0.074 to 0.613 and 0.083 to 1.0 in Kilakarsal and Vembur sheep, respectively. The heterozygosity excess ranged from 0.613 to 1.0 and 0.002 to 0.986 in Kilakarsal and Vembur sheep, respectively. In Kilakarsal sheep, the test for HWE revealed TLR9_1308_GC SNP locus with significant deviation (p<0.05) due to heterozygosity deficit. In Vembur sheep, TLR10_82_CT and TLR10_292_CG loci showed significant deviation (p<0.05) due to heterozygosity excess. Other SNP loci did not deviate from HWE (p>0.05) revealing that the population was in HWE proportions.

**Table-4:** Test for HWE at different TLR gene SNP loci in Kilakarsal and Vembur sheep.

SNP_ID	Heterozygosity deficit*		Heterozygosity excess**	
	
	Kilakarsal	Vembur	Kilakarsal	Vembur
TLR3_265_CT	0.594	0.772	0.630	0.448
TLR3_340_CT	0.574	0.083	0.720	0.986
TLR3_370_AG	0.574	0.083	0.720	0.986
TLR3_631_AG	0.574	1.000	0.720	0.473
TLR5_276_CT	0.326	1.000	0.963	0.970
TLR5_786_CT	0.580	0.839	0.691	0.375
TLR5_1354_AG	0.580	0.785	0.691	0.455
TLR5_1578_CT	0.613	0.585	0.613	0.638
TLR5_2037_CT	0.580	0.723	0.691	0.536
TLR6_49_CT	0.392	1.000	0.941	0.901
TLR6_229_GT	0.392	1.000	0.941	0.901
TLR6_589_AG	0.392	1.000	0.941	0.901
TLR6_814_AC	0.326	1.000	0.963	0.901
TLR6_1301_AG	0.558	0.723	0.774	0.536
TLR9_1308_GC	0.010*	0.883	1.000	0.300
TLR9_1769_CT	0.296	0.225	0.877	0.961
TLR9_2099_CT	0.296	0.384	0.877	0.834
TLR9_2504_CT	0.463	0.769	0.760	0.463
TLR10_82_CT	0.074	1.000	0.980	0.002**
TLR10_292_CG	0.074	1.000	0.980	0.002**
TLR10_595_AG	0.261	1.000	0.979	0.940
TLR10_771_CT	0.261	1.000	0.979	0.940
TLR10_1180_AG	nd	nd	nd	nd

* HWE p value to test for alternate hypothesis of heterozygosity deficit in sheep breeds.

** HWE p value to test for alternate hypothesis of heterozygosity excess in sheep breeds. nd: Not deducted, TLR=Toll-like receptor, SNP=Single-nucleotide polymorphism, HWE=Hardy–Weinberg equilibrium

## Discussion

### Competitive allele-specific PCR-based end-point genotyping

In this study, the SNP markers within five TLR genes (TLR3, TLR5, TLR6, TLR9 and TLR10) were utilized for genotyping of Kilakarsal and Vembur sheep through KASP-based end point genotyping. In the similar previous studies, a total of 30 SNPs in sheep major histocompatibility complex Class II and Class III regions described using KASP PCR, all SNPs exhibited Hardy–Weinberg proportions in the sheep population studied [10] and 713 sheep belonging to 22 breeds across Asia, Europe, and South America and identified 41 SNPs across 38 candidate genes were genotyped and association of genotypes with host resistance characteristics against gastrointestinal nematodes was analyzed using competitive allele-specific PCR assay based on fluorescence resonance energy transfer chemistry [11].

### Basic diversity indices

#### The gene frequency

Among 25 SNPs studied, 92% were found to be polymorphic and 8% were monomorphic in the Kilakarsal and Vembur sheep populations. The SNP TLR10_1180_AG was monomorphic (in one form) in Kilakarsal but polymorphic (more than one form) in Vembur sheep populations. A monomorphic site is one site in which all the individuals have the same form of genotype and these markers should be excluded from analysis because it gives no information. In a similar studies, out of 14 non-synonymous SNPs studied in 22 sheep breeds of different countries including Madras Red, Mecheri, and Pattanam sheep breeds of Tamil Nadu, only three of these SNPs (within TLR5, TLR7, and TLR8 genes) were found to be polymorphic, whereas the remaining 11 were monomorphic in those populations [11], and out of 16 SNPs in the genomic regions, five candidates targeted for association studies of resistance to fleece rot in Australian Merino sheep found a SNP to be monomorphic [12]. Examination of SNP loci within each breed revealed presence of both alleles in more than 90% of SNP loci, thus indicating a high degree of polymorphism and possibility of these SNP loci to be further utilized in evolutionary studies.

#### Heterozygosity

Heterozygosity is a measure of genetic variation within a population. High heterozygosity values for a breed may be due to long-term natural selection for adaptation to the mixed nature of the breeds or historic mixing of strains of different populations. A low level of heterozygosity may be due to isolation with the subsequent loss of unexploited genetic potential. Locus heterozygosity is related to the polymorphic nature of each locus. A high level of average heterozygosity at a locus could be expected to correlate with high levels of genetic variation at loci with critical importance for adaptive response to environmental changes [13]. In this study, the observed heterozygosities were estimated as 0.289 and 0.309 in Kilakarsal and Vembur sheep, respectively, whereas the expected heterozygosity values were 0.305 and 0.309 in Kilakarsal and Vembur sheep, respectively (Table-3). The findings are in agreement with the mean global observed and expected heterozygosities were 0.287 and 0.366, respectively, in the sheep breeds and mean observed heterozygosity was highest in Southwest Asian sheep populations (0.309) followed by European populations (0.296), whereas Southeast Asian populations had the least mean observed heterozygosity (0.270) [11].

#### Inbreeding coefficient (F_IS_)

The overall mean estimated inbreeding coefficient (F_IS_) was 0.107 ranging from −0.005 to 0.241 in Kilakarsal sheep breed and −0.047 ranging from −0.005 to 0.255 in Vembur sheep breed. A positive low F_IS_ values confirming that a high deficit of heterozygotes in these breeds, and if the F_IS_ is zero, then the locus is considered to be in HWE, and when F_IS_ is positive, there is a deficiency in heterozygotes and the amount of heterozygous offspring in the population will decrease, usually due to inbreeding and the mating is non-random. A number of factors such as null alleles, nature of locus, and inbreeding may lead to deficiency of heterozygotes. A negative F_IS_ value reveals an increase in heterozygosity which in turn reflects outbreeding and wide genetic variability as a result of admixture of population [9]. The estimated F_IS_ values across 23 SNP loci were found to be positive in Kilakarsal sheep except for TLR5_1578_CT and TLR9_1769_CT, whereas, in Vembur sheep population, estimated F_IS_ values found to be positive in TLR3_340_CT, TLR3_370_AG, TLR5_1578_CT, TLR9_1769_CT, and TLR9_2099_CT.

Similar result was reported with the global F_IS_ of 0.018 in the sheep population including Madras Red, Mecheri, and Pattanam sheep of Tamil Nadu [11] and the estimated mean F_IS_ values across 19 short tandem repeat loci were found to be positive in wild Punjab Urial sheep and domestic sheep from West Asia breeds except Krainer Steinschaf and Madras Red which had F_IS_ values of −0.005 and −0.001, respectively [14]. The global F_IS_ observed in the present study is much lower than the previous reports in European sheep (0.123) [15], Nigerian sheep (0.335) [16], and Bulgarian sheep (0.288) [17]. In contrast, much lower F_IS_ value of 0.024 has been reported in indigenous sheep breeds of China [18].

In this study, some SNP loci showed high F_IS_ values, namely, TLR10_292_CG and TLR10_82_CT, showed a value of 0.241 in Kilakarsal sheep, and in Vembur SNP locus, TLR3_340_CT and TLR3_370_AG showed a value of 0.255 and this reflects a high frequency of a particular allele of homozygotes (Table-3). In case of sheep, high estimates of F_IS_ can be associated with heterogeneity of flocks sampled within each breed resulting in Wahlund effect. This is also supported by the positive association of the F_IS_ estimate and the number of breeders sampled, especially when only a few animals are sampled per flock [19].

### Testing for HWE

Deviation from HWE in a population indicates possible inbreeding, population stratification, and sometimes problems with the genotyping. In populations where individuals may be affected by a particular ailment or may be under different selective pressures, these deviations can also provide evidence for association. In Kilakarsal population, the test for HWE revealed TLR9_2504_CT SNP locus with significant deviation (p<0.05) due to heterozygosity deficit. However, TLR10_82_CT and TLR10_292_CT loci in Vembur sheep populations showed a significant deviation (p<0.05) due to heterozygosity excess. Other SNP loci did not deviate from HWE (p>0.05) (Table-4).

This observation is in accordance with the previous reports where the mean number of loci deviating from HWE was 7.6, 5, 4, 7.4, and 6.3 in South Asian, Southeast Asian, Southwest Asian, European, and South American sheep, respectively, and 6, 4, and 7 in Mecheri, Madras Red, and Pattanam sheep breeds, respectively, and Hamdani sheep breed was found to be in equilibrium at panel of 41 SNP loci studied including TLR SNPs [11]. In a study with 19 STR loci, revealed 52 locus × breed combinations (21.1%) with significant deviations (p<0.05) and among these, 46 breed × locus combinations showed heterozygosity deficit (18.6%) and 6 breed × locus combinations showed heterozygosity excess (2.4%) and were found to be significantly deviate from HWE [14]. This could be due to factors such as population subdivision, selective forces operating at certain loci, null alleles, and inbreeding within sheep flocks. The heterozygote deficiencies indicate that there is strong inbreeding in these populations possibly due to unplanned and indiscriminate mating resulting in small effective population size, breeding between relatives, and consequent genetic drift, and in addition to inbreeding, another potential factor for the observed heterozygosity deficit could be natural selection forces operating at the investigated loci [20].

## Conclusion

The SNP markers within five TLR genes (TLR3, TLR5, TLR6, TLR9, and TLR10) utilized for genotyping in this study were highly polymorphic in Kilakarsal and Vembur breeds of sheep which indicated the high utility of TLR SNP loci used in this study for genetic diversity studies. This high level of average heterozygosity indicated the high levels of genetic variation at the examined loci. The overall mean F_IS_ was 0.107 in Kilakarsal sheep which revealed shortage of heterozygotes (11%) and excess of homozygotes (89%), and a number of factors such as null alleles and nature of locus may lead to deficiency of heterozygotes. In Vembur sheep, 18 loci revealed negative F_IS_ values indicating the absence of inbreeding at these loci. The majority of the loci under investigation showed significant departure from HWE which might be due to both the systematic and dispersive forces operating in the population. This study on the genetic diversity analysis of the Kilakarsal and Vembur sheep breeds revealed considerable genetic variation within the breeds and it can be utilized to improve desirable traits. The genetic information of the markers has been used as a criterion of indirect selection for genetic improvement of a quantitative trait and therefore could be useful for future breeding and *in situ* and *ex situ* conservation programs.

## Authors’ Contributions

The work was carried out by RS as part of Ph.D., Research Program, and NM, AKT, RS, GP, TPJ are the members of advisory committee. All authors read and approved the final manuscript.
